# Supplementary material for: A Likelihood Approach for Real-Time Calibration of Stochastic Compartmental Epidemic Models
Source: PLoS Comput Biol. 2017 Jan 17;13(1):e1005257. doi: 10.1371/journal.pcbi.1005257 (PMC5240920; doi:10.1371/journal.pcbi.1005257)
Supplement: S1 File — (TAR.GZ) [file pcbi.1005257.s014.tar.gz › HSPH_Online-SI-Revision/IPoi09/n1-extreme/n1-extreme_BENCH2_table-8.pdf]

1	newly 3 weeks spec	6.	8.59	{0., 22.}	170.5	1.18317	newly 3 weeks cumul	8.	14.01	{0., 40.}	187.875	1.00347	newly 5 weeks spec	31.	23.2	{0., 77.}	116.323	1.61558	newly 5 weeks cumul	58.	52.98	{0., 129.}	126.103	1.44566	newly 68 weeks cumul	923.	345.19	{0., 917.}	62.8332	1.89222
2	newly 3 weeks spec	47.	23.6	{0., 54.}	60.	0.730404	newly 3 weeks cumul	87.	51.7	{4., 121.}	60.613	0.602191	newly 5 weeks spec	79.	38.1667	{0., 79.}	54.2616	0.685163	newly 5 weeks cumul	238.	119.85	{4., 259.}	56.7857	0.642576	newly 64 weeks cumul	911.	704.817	{4., 880.}	22.7461	0.40437
3	newly 3 weeks spec	17.	21.9333	{0., 59.}	108.039	0.747908	newly 3 weeks cumul	33.	47.1333	{0., 130.}	118.182	0.675473	newly 5 weeks spec	43.	41.1667	{0., 84.}	69.7674	0.743019	newly 5 weeks cumul	103.	121.067	{0., 325.}	90.2589	0.728942	newly 66 weeks cumul	907.	633.483	{0., 900.}	30.4098	0.703197
4	newly 3 weeks spec	11.	6.63043	{0., 26.}	91.8972	1.15091	newly 3 weeks cumul	27.	15.8043	{0., 54.}	83.6554	1.02361	newly 5 weeks spec	51.	10.4783	{0., 43.}	82.0119	1.61705	newly 5 weeks cumul	107.	34.4783	{0., 121.}	79.1548	1.33487	newly 65 weeks cumul	788.	323.391	{0., 775.}	60.0806	1.52001
5	newly 3 weeks spec	48.	12.4412	{1., 29.}	74.0809	0.899153	newly 3 weeks cumul	91.	33.4706	{4., 74.}	66.7744	0.732435	newly 5 weeks spec	68.	15.0294	{2., 35.}	77.8979	0.922889	newly 5 weeks cumul	214.	62.6765	{8., 138.}	70.7119	0.802018	newly 58 weeks cumul	910.	448.676	{20., 652.}	50.6949	0.519509
6	newly 3 weeks spec	34.	55.4286	{14., 94.}	90.8613	0.368079	newly 3 weeks cumul	108.	126.5	{30., 246.}	59.3585	0.315668	newly 5 weeks spec	27.	73.2143	{34., 101.}	182.011	0.459695	newly 5 weeks cumul	166.	267.607	{96., 420.}	83.0034	0.314873	newly 63 weeks cumul	696.	800.75	{688., 891.}	17.2773	0.0687866
7	newly 3 weeks spec	32.	24.6212	{0., 92.}	91.1458	0.955478	newly 3 weeks cumul	67.	48.2121	{1., 156.}	88.3763	0.85644	newly 5 weeks spec	59.	43.1212	{0., 121.}	77.812	0.977482	newly 5 weeks cumul	173.	125.136	{1., 463.}	83.2282	0.908637	newly 67 weeks cumul	867.	619.182	{1., 912.}	31.7256	0.776614
8	newly 3 weeks spec	33.	65.1111	{23., 91.}	111.063	0.344753	newly 3 weeks cumul	77.	182.841	{60., 249.}	149.619	0.414196	newly 5 weeks spec	70.	62.4127	{34., 84.}	24.3084	0.146425	newly 5 weeks cumul	203.	310.159	{147., 408.}	65.7831	0.243987	newly 61 weeks cumul	775.	634.095	{416., 812.}	20.0655	0.115261
9	newly 3 weeks spec	10.	68.83	{0., 200.}	690.1	1.49305	newly 3 weeks cumul	20.	256.34	{0., 684.}	1280.6	1.735	newly 5 weeks spec	41.	23.67	{0., 68.}	93.9756	1.51531	newly 5 weeks cumul	80.	322.3	{0., 826.}	402.65	1.80356	newly 68 weeks cumul	770.	373.12	{0., 870.}	57.0442	1.88787
10	newly 3 weeks spec	30.	21.2444	{1., 45.}	50.6667	0.483418	newly 3 weeks cumul	68.	56.6667	{3., 120.}	57.7778	0.510014	newly 5 weeks spec	62.	25.8444	{1., 49.}	58.3871	0.630923	newly 5 weeks cumul	180.	106.933	{7., 215.}	47.9753	0.543085	newly 61 weeks cumul	869.	526.311	{27., 738.}	39.4349	0.490652
11	newly 3 weeks spec	30.	19.0725	{0., 48.}	87.2464	1.02269	newly 3 weeks cumul	41.	38.971	{0., 99.}	101.944	0.881597	newly 5 weeks spec	78.	37.6522	{0., 112.}	70.8287	1.17604	newly 5 weeks cumul	165.	105.246	{0., 293.}	78.9899	1.03175	newly 67 weeks cumul	767.	596.797	{0., 929.}	38.5541	0.904375
12	newly 3 weeks spec	6.	8.59	{0., 22.}	170.5	1.18317	newly 3 weeks cumul	12.	14.01	{0., 40.}	138.25	1.0717	newly 5 weeks spec	33.	23.2	{0., 77.}	112.545	1.63025	newly 5 weeks cumul	59.	52.98	{0., 129.}	124.881	1.44967	newly 68 weeks cumul	724.	345.19	{0., 917.}	65.8494	1.83857
13	newly 3 weeks spec	17.	41.6415	{7., 69.}	168.257	0.470406	newly 3 weeks cumul	47.	107.849	{14., 183.}	153.312	0.43475	newly 5 weeks spec	37.	50.6038	{14., 82.}	62.6721	0.320343	newly 5 weeks cumul	107.	206.547	{47., 335.}	115.853	0.388858	newly 61 weeks cumul	729.	664.358	{458., 803.}	15.2341	0.164148
14	newly 3 weeks spec	25.	73.6094	{19., 120.}	207.438	0.487602	newly 3 weeks cumul	67.	162.5	{35., 279.}	162.127	0.428735	newly 5 weeks spec	83.	91.3594	{42., 133.}	36.0505	0.178528	newly 5 weeks cumul	206.	340.5	{114., 501.}	82.4939	0.283593	newly 65 weeks cumul	782.	812.797	{674., 910.}	13.8687	0.0631819
15	newly 3 weeks spec	26.	29.1091	{5., 55.}	59.7902	0.34266	newly 3 weeks cumul	51.	74.3273	{10., 148.}	78.9661	0.327851	newly 5 weeks spec	70.	38.6545	{15., 66.}	45.4545	0.398032	newly 5 weeks cumul	147.	148.745	{34., 270.}	45.8132	0.274334	newly 60 weeks cumul	692.	673.	{540., 809.}	12.8875	0.102692
16	newly 3 weeks spec	24.	16.1316	{0., 69.}	95.0658	1.16295	newly 3 weeks cumul	40.	33.5789	{0., 113.}	101.974	1.10951	newly 5 weeks spec	42.	26.6579	{0., 92.}	86.4035	1.21755	newly 5 weeks cumul	120.	83.2105	{0., 331.}	92.6096	1.27253	newly 67 weeks cumul	755.	474.934	{0., 888.}	47.4329	1.29296
17	newly 3 weeks spec	26.	10.3636	{0., 28.}	70.2098	0.811421	newly 3 weeks cumul	47.	24.6	{2., 62.}	66.6151	0.664838	newly 5 weeks spec	50.	16.3273	{0., 46.}	71.7091	0.965657	newly 5 weeks cumul	121.	53.8182	{4., 138.}	68.1443	0.75015	newly 64 weeks cumul	819.	563.727	{4., 796.}	31.4486	0.514577
18	newly 3 weeks spec	13.	50.7164	{0., 147.}	345.465	0.907069	newly 3 weeks cumul	23.	123.373	{0., 362.}	486.502	0.991547	newly 5 weeks spec	42.	57.3582	{0., 119.}	103.802	0.879513	newly 5 weeks cumul	99.	236.925	{0., 616.}	197.935	0.964472	newly 67 weeks cumul	912.	611.94	{0., 898.}	32.9929	0.845412
19	newly 3 weeks spec	14.	41.04	{0., 146.}	307.571	1.40375	newly 3 weeks cumul	34.	109.	{0., 441.}	336.294	1.52175	newly 5 weeks spec	39.	31.55	{0., 119.}	102.385	1.47624	newly 5 weeks cumul	99.	178.83	{0., 672.}	196.273	1.69207	newly 68 weeks cumul	880.	384.65	{0., 898.}	57.2739	1.91275
20	newly 3 weeks spec	50.	45.9344	{11., 78.}	44.459	0.322144	newly 3 weeks cumul	121.	112.377	{27., 195.}	46.7958	0.326194	newly 5 weeks spec	81.	62.4918	{22., 92.}	30.2975	0.275967	newly 5 weeks cumul	273.	228.951	{76., 354.}	35.0748	0.291769	newly 61 weeks cumul	893.	742.41	{674., 849.}	16.8634	0.178745
21	newly 3 weeks spec	10.	28.9833	{1., 66.}	226.167	0.612009	newly 3 weeks cumul	39.	64.3167	{6., 142.}	117.051	0.480426	newly 5 weeks spec	27.	44.9	{0., 86.}	108.765	0.544255	newly 5 weeks cumul	84.	147.1	{6., 315.}	118.175	0.493688	newly 64 weeks cumul	711.	712.133	{7., 896.}	23.835	0.338578
22	newly 3 weeks spec	50.	52.0167	{0., 110.}	70.6333	0.615907	newly 3 weeks cumul	92.	120.733	{0., 243.}	89.7101	0.630835	newly 5 weeks spec	80.	63.2	{0., 110.}	45.2917	0.613008	newly 5 weeks cumul	222.	243.767	{0., 458.}	68.1231	0.635103	newly 65 weeks cumul	825.	620.283	{0., 873.}	28.2848	0.590065
23	newly 3 weeks spec	21.	12.2676	{0., 38.}	92.2871	1.15606	newly 3 weeks cumul	38.	24.3662	{0., 77.}	91.0304	1.02142	newly 5 weeks spec	50.	25.7465	{0., 94.}	82.3099	1.27125	newly 5 weeks cumul	115.	69.6901	{0., 249.}	86.2829	1.1596	newly 67 weeks cumul	744.	480.465	{0., 892.}	47.1736	1.18633
24	newly 3 weeks spec	11.	8.59	{0., 22.}	116.273	1.31705	newly 3 weeks cumul	16.	14.01	{0., 40.}	115.563	1.13084	newly 5 weeks spec	41.	23.2	{0., 77.}	101.756	1.68413	newly 5 weeks cumul	85.	52.98	{0., 129.}	103.835	1.53823	newly 68 weeks cumul	886.	345.19	{0., 917.}	62.3059	1.87883
25	newly 3 weeks spec	21.	13.1509	{0., 33.}	71.8778	0.900375	newly 3 weeks cumul	34.	31.6226	{0., 72.}	80.6881	0.758516	newly 5 weeks spec	31.	19.8302	{0., 54.}	69.0201	0.814351	newly 5 weeks cumul	93.	67.0943	{0., 143.}	68.7158	0.832879	newly 65 weeks cumul	758.	505.642	{0., 846.}	39.0974	0.891513
26	newly 3 weeks spec	12.	13.2113	{0., 31.}	119.249	0.95307	newly 3 weeks cumul	15.	24.1549	{1., 59.}	142.535	0.65588	newly 5 weeks spec	48.	28.8028	{0., 96.}	88.2336	1.23036	newly 5 weeks cumul	84.	73.6901	{1., 191.}	99.3293	0.912578	newly 67 weeks cumul	923.	489.803	{1., 915.}	47.1991	1.09968
27	newly 3 weeks spec	38.	32.6905	{5., 65.}	47.5564	0.462047	newly 3 weeks cumul	98.	96.2857	{20., 182.}	50.3887	0.345143	newly 5 weeks spec	56.	33.4286	{0., 76.}	53.1463	0.709504	newly 5 weeks cumul	192.	162.976	{34., 299.}	43.2168	0.361114	newly 62 weeks cumul	795.	389.238	{35., 751.}	51.8538	0.580586
28	newly 3 weeks spec	39.	85.4762	{13., 153.}	145.95	0.525577	newly 3 weeks cumul	74.	173.508	{27., 335.}	161.755	0.560949	newly 5 weeks spec	88.	106.413	{32., 153.}	45.6349	0.340955	newly 5 weeks cumul	201.	386.921	{83., 585.}	116.22	0.523778	newly 66 weeks cumul	916.	787.095	{571., 934.}	14.7692	0.293186
29	newly 3 weeks spec	8.	14.4091	{1., 37.}	148.295	0.592695	newly 3 weeks cumul	18.	32.5909	{2., 85.}	143.687	0.511297	newly 5 weeks spec	61.	19.9545	{0., 53.}	67.5112	1.00518	newly 5 weeks cumul	116.	70.1591	{4., 173.}	63.1074	0.639473	newly 63 weeks cumul	930.	532.25	{4., 810.}	42.7688	0.564227
30	newly 3 weeks spec	51.	61.5152	{43., 83.}	33.3928	0.16923	newly 3 weeks cumul	155.	193.061	{134., 253.}	36.5396	0.160057	newly 5 weeks spec	44.	52.4091	{27., 79.}	39.635	0.174097	newly 5 weeks cumul	251.	305.106	{209., 392.}	31.9751	0.140637	newly 58 weeks cumul	605.	582.152	{371., 792.}	21.6829	0.103112
31	newly 3 weeks spec	24.	28.8028	{0., 96.}	125.059	1.08169	newly 3 weeks cumul	52.	62.7465	{0., 181.}	124.242	1.07321	newly 5 weeks spec	60.	36.2535	{0., 106.}	74.9296	1.22879	newly 5 weeks cumul	138.	131.93	{0., 396.}	102.092	1.21721	newly 67 weeks cumul	874.	478.859	{0., 884.}	46.2291	1.31282
32	newly 3 weeks spec	21.	43.127	{0., 108.}	160.695	0.681983	newly 3 weeks cumul	42.	79.873	{1., 187.}	146.561	0.615311	newly 5 weeks spec	41.	83.7143	{0., 168.}	141.502	0.687275	newly 5 weeks cumul	113.	228.571	{1., 513.}	144.697	0.666904	newly 67 weeks cumul	845.	715.841	{1., 910.}	21.9987	0.519004
33	newly 3 weeks spec	14.	3.89474	{0., 9.}	74.0602	0.947277	newly 3 weeks cumul	41.	10.9474	{0., 37.}	74.5828	1.051	newly 5 weeks spec	35.	4.68421	{0., 16.}	86.6165	1.419	newly 5 weeks cumul	96.	19.5263	{0., 68.}	79.6601	1.21923	newly 63 weeks cumul	860.	212.395	{0., 557.}	75.3029	1.42276
34	newly 3 weeks spec	20.	28.2656	{0., 65.}	106.797	0.653201	newly 3 weeks cumul	48.	54.3438	{2., 122.}	86.9141	0.579941	newly 5 weeks spec	70.	51.125	{0., 119.}	56.6518	0.724323	newly 5 weeks cumul	151.	145.578	{2., 323.}	71.7819	0.628918	newly 66 weeks cumul	845.	709.406	{2., 929.}	22.9327	0.498966
35	newly 3 weeks spec	18.	7.72603	{0., 22.}	92.5419	1.37985	newly 3 weeks cumul	50.	16.6164	{0., 44.}	87.3699	1.32358	newly 5 weeks spec	58.	14.3425	{0., 40.}	87.7893	1.6926	newly 5 weeks cumul	143.	42.6301	{0., 114.}	88.8878	1.56594	newly 67 weeks cumul	834.	342.521	{0., 868.}	60.816	1.62446
36	newly 3 weeks spec	24.	24.0392	{2., 54.}	68.6275	0.468134	newly 3 weeks cumul	59.	54.6275	{11., 116.}	62.2466	0.421298	newly 5 weeks spec	53.	35.9804	{3., 72.}	49.5006	0.478455	newly 5 weeks cumul	146.	121.059	{19., 242.}	55.6809	0.438899	newly 64 weeks cumul	848.	657.529	{299., 851.}	22.7063	0.315223
37	newly 3 weeks spec	45.	19.1395	{3., 42.}	58.8114	0.580582	newly 3 weeks cumul	72.	51.6512	{9., 117.}	56.1047	0.435811	newly 5 weeks spec	59.	24.5349	{6., 48.}	60.3074	0.587245	newly 5 weeks cumul	179.	99.3256	{20., 215.}	53.1116	0.484654	newly 59 weeks cumul	848.	551.814	{320., 709.}	34.9276	0.315344
38	newly 3 weeks spec	27.	46.4127	{9., 94.}	108.583	0.426108	newly 3 weeks cumul	70.	107.619	{23., 230.}	95.6463	0.391442	newly 5 weeks spec	62.	59.6984	{15., 99.}	39.0425	0.31194	newly 5 weeks cumul	186.	224.016	{60., 408.}	63.4835	0.357291	newly 64 weeks cumul	878.	753.508	{652., 877.}	14.5117	0.187401
39	newly 3 weeks spec	9.	11.6712	{0., 32.}	159.056	1.19529	newly 3 weeks cumul	17.	24.8493	{0., 72.}	163.336	1.16036	newly 5 weeks spec	28.	16.5753	{0., 53.}	103.131	1.49425	newly 5 weeks cumul	66.	55.7671	{0., 153.}	119.24	1.46012	newly 67 weeks cumul	893.	339.151	{0., 854.}	62.1869	1.79767
40	newly 3 weeks spec	58.	72.9194	{27., 117.}	58.0367	0.276953	newly 3 weeks cumul	138.	164.081	{40., 286.}	57.3282	0.301542	newly 5 weeks spec	45.	88.6452	{47., 125.}	109.247	0.373852	newly 5 weeks cumul	245.	338.597	{134., 506.}	61.0599	0.282928	newly 65 weeks cumul	775.	794.548	{671., 914.}	14.693	0.123499
41	newly 3 weeks spec	31.	18.6667	{0., 46.}	58.4229	0.762366	newly 3 weeks cumul	62.	67.8889	{4., 131.}	72.3417	0.48518	newly 5 weeks spec	37.	18.2963	{0., 52.}	64.965	0.954743	newly 5 weeks cumul	130.	103.444	{6., 217.}	57.2934	0.527301	newly 60 weeks cumul	655.	211.222	{6., 502.}	68.2386	0.874727
42	newly 3 weeks spec	20.	15.77	{0., 37.}	120.45	1.43798	newly 3 weeks cumul	35.	28.06	{0., 65.}	117.6	1.41711	newly 5 weeks spec	52.	28.69	{0., 112.}	96.9423	1.68706	newly 5 weeks cumul	108.	79.95	{0., 251.}	112.528	1.67605	newly 68 weeks cumul	918.	343.48	{0., 914.}	62.8671	1.9732
43	newly 3 weeks spec	9.	6.31579	{0., 16.}	95.614	0.969265	newly 3 weeks cumul	17.	12.9079	{0., 35.}	86.1455	0.772288	newly 5 weeks spec	64.	16.1316	{0., 69.}	80.8799	1.46593	newly 5 weeks cumul	106.	40.1711	{0., 134.}	80.5983	1.15775	newly 67 weeks cumul	902.	481.526	{0., 894.}	46.9541	1.14715
44	newly 3 weeks spec	40.	12.0714	{0., 35.}	74.5536	1.05003	newly 3 weeks cumul	67.	25.3036	{1., 67.}	71.1887	0.877073	newly 5 weeks spec	68.	16.8929	{0., 44.}	76.9433	1.25788	newly 5 weeks cumul	179.	56.9107	{1., 138.}	72.4561	1.00863	newly 65 weeks cumul	873.	517.768	{1., 854.}	40.9528	0.888688
45	newly 3 weeks spec	11.	14.87	{0., 47.}	183.364	1.35602	newly 3 weeks cumul	17.	24.67	{0., 71.}	182.294	1.21983	newly 5 weeks spec	50.	23.5	{0., 116.}	102.52	1.79028	newly 5 weeks cumul	90.	69.56	{0., 258.}	127.467	1.60048	newly 68 weeks cumul	926.	333.55	{0., 904.}	64.0335	1.94433
46	newly 3 weeks spec	19.	4.04348	{0., 12.}	82.151	1.36544	newly 3 weeks cumul	34.	10.3696	{0., 30.}	75.7673	1.21675	newly 5 weeks spec	55.	6.06522	{0., 20.}	88.9723	1.80014	newly 5 weeks cumul	129.	21.0435	{0., 64.}	83.6872	1.58239	newly 65 weeks cumul	889.	252.63	{0., 759.}	71.5826	1.78448
47	newly 3 weeks spec	19.	25.5	{0., 67.}	107.895	0.69016	newly 3 weeks cumul	29.	49.4063	{1., 122.}	133.082	0.630287	newly 5 weeks spec	39.	49.9531	{0., 116.}	98.1971	0.747682	newly 5 weeks cumul	94.	136.672	{3., 310.}	111.652	0.662686	newly 66 weeks cumul	840.	710.141	{3., 923.}	22.3605	0.485162
48	newly 3 weeks spec	17.	115.544	{39., 170.}	587.719	0.85748	newly 3 weeks cumul	48.	282.947	{76., 458.}	498.319	0.800729	newly 5 weeks spec	41.	103.93	{60., 156.}	161.018	0.474466	newly 5 weeks cumul	126.	503.754	{275., 674.}	306.823	0.686803	newly 66 weeks cumul	757.	767.877	{577., 910.}	16.6632	0.194047
49	newly 3 weeks spec	30.	21.39	{0., 76.}	125.1	1.6465	newly 3 weeks cumul	64.	43.45	{0., 152.}	122.641	1.67643	newly 5 weeks spec	69.	25.35	{0., 115.}	90.4203	1.85689	newly 5 weeks cumul	166.	92.3	{0., 399.}	108.169	1.86045	newly 68 weeks cumul	950.	331.03	{0., 897.}	65.1547	2.07685
50	newly 3 weeks spec	39.	43.9828	{10., 87.}	58.9302	0.334969	newly 3 weeks cumul	92.	218.603	{78., 388.}	146.683	0.399758	newly 5 weeks spec	56.	16.9483	{1., 36.}	72.9372	0.831165	newly 5 weeks cumul	194.	264.655	{99., 488.}	62.9221	0.275032	newly 66 weeks cumul	717.	296.345	{100., 567.}	58.8563	0.506781
